# Examining the impact of substance use on hospital length of stay in schizophrenia spectrum disorder: a retrospective analysis

**DOI:** 10.1186/s12916-024-03447-3

**Published:** 2024-06-10

**Authors:** Achim Burrer, Stephan T. Egger, Tobias R. Spiller, Matthias Kirschner, Philipp Homan, Erich Seifritz, Stefan Vetter

**Affiliations:** 1 Department of Psychiatry, Psychotherapy and Psychosomatics, Psychiatric Hospital, University of Zurich, Zurich, Switzerland; 2 Division of Adult Psychiatry, Department of Psychiatry, Geneva University Hospitals, Geneva, Switzerland; 3 Neuroscience Center Zurich, University of Zurich, Zurich, Switzerland

**Keywords:** Schizophrenia, Length of stay, Substance use, Hospitalization

## Abstract

**Background:**

Among patients diagnosed with schizophrenia, the presence of substance use poses an aggravating comorbidity, exerting a negative impact on the course of the disease, adherence to therapeutic regimens, treatment outcomes, duration of hospital stays, and the frequency of hospitalizations. The primary objective of the present study is to investigate the relationship between comorbid substance use disorders, antipsychotic treatment, and the length of stay in individuals hospitalized for treatment of schizophrenia.

**Methods:**

We conducted a retrospective analysis of electronic health records spanning a 12-month period, specifically focusing on adult patients diagnosed with schizophrenia who were discharged from the University Hospital of Psychiatry Zurich between January and December 2019. We documented the number and types of diagnosed substance use disorder, the antipsychotic treatment, the length of stay, and the number of previous hospitalizations for each patient.

**Results:**

Over a third (*n* = 328; 37.1%) of patients with schizophrenia had comorbid substance use with cannabis being the most frequent consumed substance. Patients with substance use (either single or multiple) were more frequently hospitalized; those with multiple substance use more frequently than those with a single substance use (*F*(2, 882) = 69.06; *p* < 0.001). There were no differences regarding the rate of compulsory admission. Patients with no substance use had a lower HoNOS score at discharge (*F*(2, 882) = 4.06). Patients with multiple substance use had a shorter length of stay (*F*(2, 882) = 9.22; *p* < 0.001), even after adjusting for duration of illness, previous hospitalizations, diagnosis, and antipsychotic treatment.

**Conclusions:**

In patients with schizophrenia, comorbid single or multiple substance use has a relevant negative impact on treatment and thus on the course of disease. Substance use in patients with schizophrenia should therefore receive special attention in order to reduce re-hospitalization rates and improve the clinical outcome.

## Background

Substance use poses a significant challenge for the field of public mental health [1, 2] by leading to a poorer outcome and prognosis of other concomitant psychiatric disorders. Moreover, research has shown varying effects of substance use disorders (SUDs) on hospitalization lengths among patients with schizophrenia spectrum disorder (SSD). In detail comorbid cocaine use [3] and alcohol use disorder [4] in patients with SSD have been associated with shorter hospital stays. Generally, patients with SSD and a co-occurring SUD have shorter median hospital stays compared to those without a dual diagnosis [5]. The general population of individuals with SSD with cannabis use disorder have shorter hospital stays compared to those without a cannabis use disorder [6, 7]. In contrast individuals with SSD and concurrent cannabis use disorder interestingly tend to experience extended durations of stay during their initial hospitalization [8].

Besides the general effect that SUD are associated with shorter hospitalization (i.e., length of stay), individuals with SSD and SUD have therefore been associated with a greater number of re-hospitalizations and thus with a higher number of cumulative hospitalization days [9, 10].

Moreover, SUD in SSD is associated to further issues: Firstly, compulsory hospitalizations among psychiatric patients with a co-occurring SUD increased over time [11]. Secondly, comorbid substance use disorder has been associated with an increased risk of violent crime in individuals with schizophrenia [12]. Thirdly, among individuals with SSD those with comorbid SUD are also exposed to an increased risk of psychosocial problems such as occupational problems, housing problems, and economic problems [13].

Understanding the relationship between comorbid substance use and length of stay in individuals with schizophrenia is therefore crucial for developing effective treatment strategies and improving outcomes for this population. The variations in hospitalization due to SUDs present challenges for healthcare providers, authorities, and cost payers in treating patients with SSD and a comorbid SUD.

We know that SUD in SSD tend to be related to shorter LOS but at the same time SUDs are related to factors such as involuntary stay, violence, poorer treatment adherence, and higher relapse rates potentially leading to increase re-hospitalization. However, the specific impact of multiple SUDs on the course of treatment, especially regarding the LOS, remains unclear. Therefore, this retrospective study aims to address this knowledge gap by closely examining the effects of both single and multiple comorbid SUDs on key aspects of hospitalization, including the LOS and the frequency of hospital admissions. Beyond, the presence of a SUD might also affect the type of antipsychotic treatment as we could show in a previous investigation. Here, patients receiving a long-acting injectable were more likely to be diagnosed with a comorbid substance use disorder [14].

Since there is data that SUDs in patients with SSD can have both a prolonging and a shortening effect on the LOS, this retrospective study is exploratory in design and no hypothesis is presented.

## Methods

### Study design and sample

The present study was designed as retrospective analysis of the length and frequency of hospital treatment in patients with SSD with particular emphasis on the interplay with comorbid SUD. The study drew upon data collected from adult patients of the Department of Psychiatry, Psychotherapy, and Psychosomatics of the University Hospital of Psychiatry Zurich, spanning a comprehensive timeframe of 12 months, specifically from January 1st to December 31st, 2019. The project was approved by the Ethics Committee of the Canton of Zurich (BASEC-Nr. Req-2021–00376). Patient consent was waived due to approval by the Ethics Committee.

The University Hospital of Psychiatry Zurich serves as a psychiatric care provider, offering inpatient and outpatient treatments in the vicinity of Zurich, Switzerland. Its service mandate encompasses a diverse catchment area, including both urban and rural regions, serving approximately 500,000 residents. The hospital maintains an electronic health record system.

### Clinical assessment, diagnosis, and data extraction

Attending psychiatrists, psychiatry residents, or clinical psychologists completed the rating through a clinical interview and observation process. The raters had a standardized introduction to the rating scales. Psychiatric diagnoses were made by the case responsible clinician according to the International Classification of Disease 10 (Diagnostic Criteria for Schizophrenia [ICD-10] chapter F2) and validated by a board-certified senior psychiatrist.

The focus of our study was on patients with SSD, as defined by the ICD-10. Consequently, we included patients who were diagnosed with schizophrenia (F20), schizotypal disorder (F21), delusional disorder (F22), brief psychotic episode (F23), or schizoaffective disorder (F25). All subgroups of the respective ICD-10 diagnoses were included. Patients diagnosed with purely substance-induced psychosis (ICD-10 chapter F1) not meeting the criteria of SSD were not included in the analysis.

In addition, we included concomitant substance use disorders according to the ICD-10 (i.e., alcohol use disorder: F10; opioid use disorder: F11; cannabis use disorder: F12; benzodiazepine use disorder: F13; cocaine use disorder: F14; and stimulant use disorder: F15), without further distinguishing between intoxication, harmful use, dependence, and withdrawal. For the analysis, we extracted basic demographic information such as age, sex, education, and marital status from the electronic health record. We also included clinical variables as the Health of the Nation Outcome Scales (HoNOS) to assess severity and change.

We recorded the number of past hospitalizations of patients. For the current admission, we recorded if patients were admitted voluntarily or by compulsorily admission order. We determined the hospital length of stay (LOS) (i.e., the time elapsed from admission to discharge). As a correlate for the duration of illness, we calculated the time elapsed from the first admission. We retrieved the antipsychotic treatment regarding if it was prescribed as a mono- or combination therapy; we also assessed if antipsychotics were prescribed as a long-acting injectable formulation. Finally, we also determined if the discharge was regular or against medical advice (including those discharged by court mandate or those that leaved unnoticed the hospital).

### Outcome measures

To evaluate the clinical severity of psychiatric disorders, we employed the Health of the Nation Outcome Scales (HoNOS). The HoNOS is a standardized measurement tool designed to assess the severity and impact of psychiatric conditions across 12 distinct domains. Each domain is represented by an item and is rated on a Likert-like scale, ranging from “0” (indicating the absence of a problem) to “4” (representing a severe to very severe problem). The HoNOS can be analyzed both globally, providing an overall assessment (sum score ranging from 0 to 48), or at the individual item level, allowing for a more detailed examination of specific areas [15]. HoNOS was rated at admission and discharge by the treating physician or psychologist and subsequently validated by a board-certified psychiatrist as this is the standard procedure for HoNOS rating in our hospital.

### Statistical analysis

We subdivided the sample according to the presence of substance use and the number of different substances used disregarding the severity or pattern of consumption of each substance. Therefore, we defined three groups: no substance use, single substance use, and multiple substance use.

To present the baseline demographic and clinical characteristics of the sample, we used descriptive statistics such as mean, standard deviation, median, and percentages. We checked the assumptions for parametric testing. To examine the group differences in the continuous variables, we used analysis of variance (ANOVA); for significant results we conducted subsequent pairwise comparisons using the Student’s *t*-test. To evaluate differences in categorical variables, we used the chi-square test. For significant results, a chi-square omnibus comparison followed, through the analysis of residuals, we calculated the percentage that each category contributes to the overall chi-squared score. We calculated the odds ratios (OR) for several outcomes, providing additional insights into the relationships and associations observed. The odds ratios (OR) were adjusted using logistic regression for potential baseline confounders such as age, sex, education, marital status, and service use parameters such as admission status and length of stay. We utilized Kaplan–Meier time-to-event curves to visualize and analyze the hospital length of stay and time to readmission. To assess the statistical significance of the observed differences, we employed the log-rank test and determined the corresponding *p* value.

All significance tests were conducted as two-tailed tests, with a predetermined level of significance set at *p* < 0.05. Additionally, effect sizes were evaluated using eta-square for continuous variables and phi for categorical variables.

All statistical analyses were performed using RStudio (2023.12.1 + 402), R (version 4.3.2). With the packages: tidyverse (2.0.0); rstatix (0.7.2); moments (0.14.1); survival (3.5–7); survminer (0.4.9); adjustedCurves (0.10.1); riskRegression (2023.12.21).

## Results

### Sample characteristics

During the observation period, a total of 2203 patients were admitted, out of which 885 individuals (40.2%) had a diagnosis of SSD and were included in the final analysis. The mean age of the patients was 40.51 years (SD = 12.44). Among the included patients, the majority were male (*n* = 557, 62.9%), unmarried (*n* = 318, 35.9%), and held a high school diploma as their highest vocational degree (*n* = 570, 64.4%). Notably, patients with a SUD, using single or involving multiple substances, were younger compared to those without a SUD (*F*(2, 882) = 7.233, *p* = 0.016).

The average time lapse since the first hospitalization (as a surrogate for duration of illness) was 8.71 years (SD = 8.05). Patients had an average of 10.63 (SD = 15.25) previous hospitalizations, with a right-tailed distribution. At the time of admission, the patients had an average HoNOS score of 20.85 (7.77). The length of hospital stay had an average of 24.62 days (SD = 27.13) and exhibited a right-tailed (skewness: 2.50; kurtosis: 10.88) distribution.

For further details, please refer to Table 1.

**Table 1:** Demographic characteristics of the sample according to the presence of a SUD

	**No SUD**	**Single SUD**	**Multiple SUD**		
	***n*** **= 557**	***n*** **= 171**	***n*** **= 157**	Statistic	*p*
	*Mean (SD)*	*Mean (SD)*	*Mean (SD)*		
**Age**	41.71 (12.72)^a^	38.13 (13.00)^a^	38.83 (10.13)^a^	*F*(2, 882) = 7.233	0.016
	*n (%)*	*n (%)*	*n (%)*		
**Sex**					
Female	238 (42.7)^a^	47 (27.5)^a^	43 (27.4)^a^	*X* ^2^(2, 885) = 20.69	< 0.001
Male	319 (57.3)	124 (72.5)	114 (72.6)	*X* ^2^(2, 885) = 20.69	< 0.001
**Education**					
Regular education	339 (60.8)	97 (56.7)	134 (85.4)	*X* ^2^(2, 885) = 37.46	< 0.001
Apprenticeship	127 (22.8)	44 (25.7)	14 (8.9)	*X* ^2^(2, 885) = 17.26	< 0.001
College/university	91 (16.4)	30 (17.6)	9 (5.7)	*X* ^2^(2, 885) = 12.37	0.002
**Marital status**					
Single	194 (34.8)	58 (33.9)	66 (42.0)	*X* ^2^(2, 885) = 3.12	0.20
Married	93 (16.7)	28 (16.4)	18 (11.5)	*X* ^2^(2, 885) = 2.60	0.27
Unmarried/unknown	270 (48.5)	85 (49.7)	73 (46.5)	*X* ^2^(2, 885) = 0.34	0.84

^a^Single and multiple substance use < no substance use

### Diagnoses and hospital treatment

**Fig. 1:**
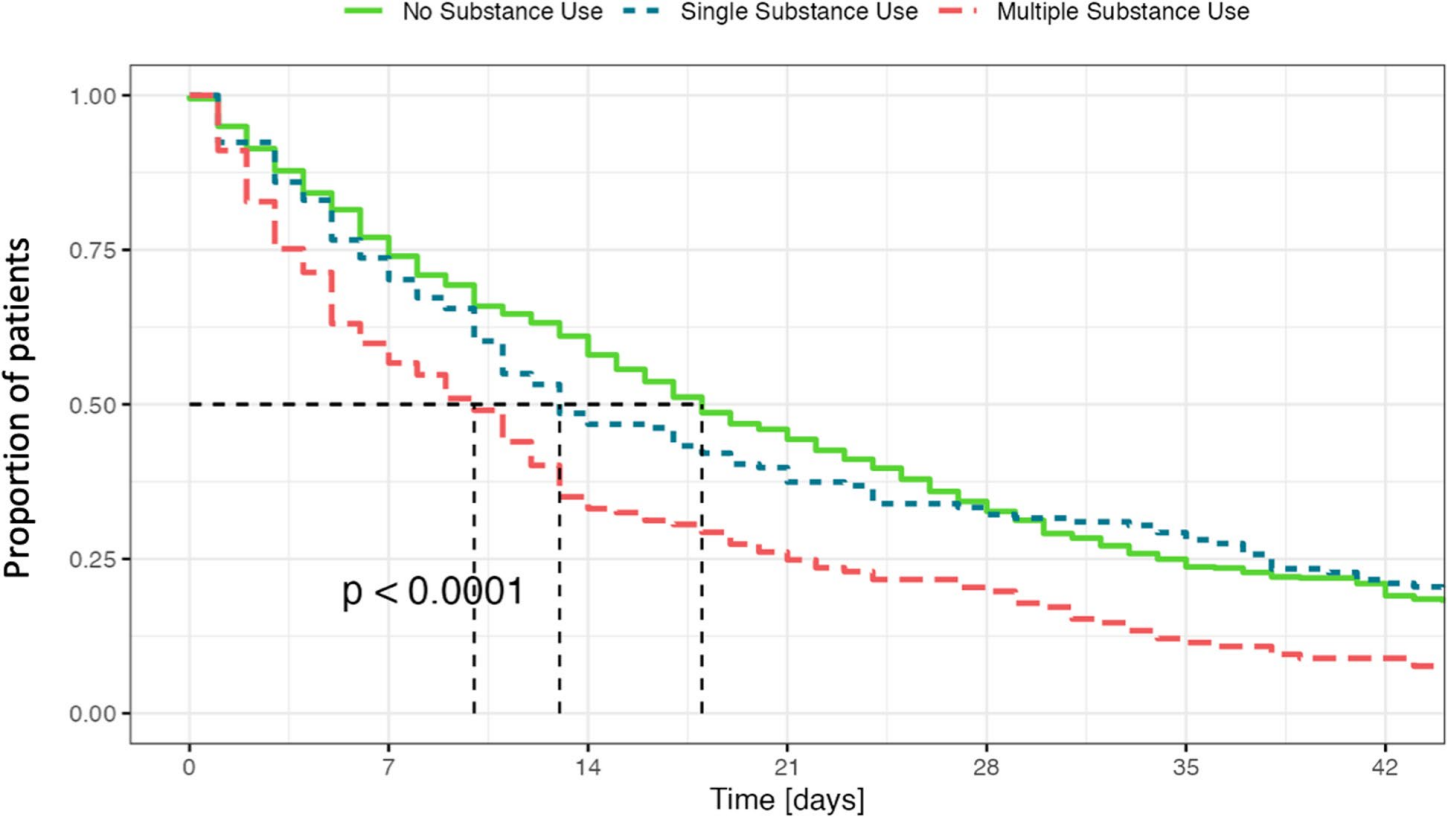
Length of stay according to the presence of a substance use disorder. Kaplan–Meier plot of length of stay in patients without SUD (green), single SUD (blue), and multiple SUD (red)

For more comprehensive information, please refer to Table 2 and Fig. 1. Among the patients included in the study, approximately two-thirds (*n* = 586, 66.2%) received a diagnosis of schizophrenia. Schizoaffective disorder accounted for nearly one-fifth of the cases (*n* = 156, 17.6%), followed by brief psychotic episodes (*n* = 117, 13.2%) and delusional disorder (*n* = 26, 3.0%).

**Table 2:** Clinical characteristics of the sample according to the presence of a substance use disorder

	**No SUD**	**Single SUD**	**Multiple SUD**		
	***n*** **= 557**	***n*** **= 171**	***n*** **= 157**	Statistic	*p*
	*n (%)*	*n (%)*	*n (%)*		
**Diagnosis**					
Brief psychotic disorder	88 (15.8)	18 (10.5)	11 (7.0)	*X* ^2^(2, 885) = 9.59	0.008
Schizophrenia	345 (61.9)	110 (64.3)	131 (83.4)	*X* ^2^(2, 885) = 25.65	< 0.001
Delusional disorder	20 (3.6)	5 (2.9)	1 (0.6)	*X* ^2^(2, 885) = 3.74	0.15
Schizoaffective disorder	104 (18.7)	38 (22.2)	14 (8.9)	*X* ^2^(2, 885) = 11.11	0.004
**Substance use disorder**					
Alcohol	–	36 (21.1)	60 (38.2)	*X* ^2^(1, 571) = 2.71	0.09
Amphetamine	–	14 (8.2)	27 (17.2)	*X* ^2^(1, 571) = 0.19	0.06
Benzodiazepine	–	21 (12.3)	40 (25.5)	*X* ^2^(1, 571) = 0.44	0.50
Cannabis	–	85 (49.7)	114 (72.6)	*X* ^2^(1, 571) = 22.80	< 0.001
Cocaine	–	7 (4.1)	96 (61.1)	*X* ^2^(1, 571) = 30.77	< 0.001
Opiate	–	8 (4.7)	63 (40.4)	*X* ^2^(1, 571) = 12.49	< 0.001
	*Mean (SD)*	*Mean (SD)*	*Mean (SD)*		
Duration of illness	93.48 (95.14)^a^	103.20 (96.73)^a^	145.25 (90.87)^a^	*F*(2, 882) = 18.32	< 0.001
Number of hospitalization	6.93 (9.16)^b^	12.42 (15.94)^b^	21.83 (23.76)^b^	*F*(2, 882) = 69.06	< 0.001
	*n (%)*	*n (%)*	*n (%)*		
Compulsive admission	281 (50.4)	98 (57.3)	94 (59.8)	*X* ^2^(2, 885) = 5.64	0.06
Regular discharge	493 (88.5)	156 (91.2)	120 (76.4)	*X* ^2^(2, 885) = 2.84	0.24
	*Mean (SD)*	*Mean (SD)*	*Mean (SD)*		
HoNOS (12 items)					
HoNOS admission	20.36 (7.54)	21.50 (6.91)	24.71 (10.72)	*F*(2, 882) = 2.62	0.08
HoNOS discharge	12.69 (7.06)^b^	16.25 (8.13)	15.47 (10.41)	*F*(2, 882) = 4.06	0.02
HoNOS difference	7.67 (8.54)	7.25 (6.86)	9.24 (8.96)	*F*(2, 882) = 1.64	0.19
HoNOS (without item 3)					
HoNOS admission	19.65 (7.25)	19.39 (6.55)	21.35 (10.70)	*F*(2, 882) = 0.457	0.64
HoNOS discharge	12.33 (7.00)	14.72 (7.45)	13.71 (9.33)	*F*(2, 882) = 1.78	0.17
HoNOS difference	7.32 (7.11)	6.67 (6.08)	7.65 (7.02)	*F*(2, 882) = 1.74	0.18
Length of stay	26.18 (26.02)^c^	27.16 (34.08)^c^	16.31 (20.18)^c^	*F*(2, 882) = 9.22	< 0.001

Percentages of substances may add up to over 100% because individual patients may have consumed more than one substance

^a^No and single substance use < multiple substance use

^b^No substance use < single substance use < multiple substance use

^c^No and single substance use > multiple substance use

In terms of comorbid substance use, approximately one-third of the patients (*n* = 328, 37.1%) had at least one SUD. Among those with SUD, almost half (*n* = 157, 47.9%) exhibited a SUD involving two or more substances. The most prevalent substances among patients with SUD were cannabis (*n* = 199, 60.7%), followed by cocaine (*n* = 103, 31.4%), alcohol (*n* = 96, 29.2%), opioids (*n* = 71, 21.6%), benzodiazepines (*n* = 61, 18.6%), and amphetamines (*n* = 41, 12.5%).

Patients with SUD were predominantly male, constituting almost three-quarters of the group, in contrast to those without SUD (χ^2^(2, 885) = 20.69; *p* < 0.001). Notably, patients with a SUD, using single or involving multiple substances, were younger compared to those without a SUD (*F*(2, 882) = 7.233, *p* = 0.016). Additionally, patients with multiple SUD had lower education levels compared to those with single or no SUD (χ^2^(4, 885) = 37.55; *p* < 0.001). No significant differences regarding marital status were observed.

Patients with multiple SUD exhibited a longer duration of illness compared to those with no or single SUD (*F*(2, 882) = 18.32; *p* < 0.001). Furthermore, patients without SUD had a lower frequency of hospitalizations compared to those with SUD (either single or multiple), while patients with multiple SUD had a higher frequency of hospitalizations than those with single SUD (*F*(2, 882) = 69.06; *p* < 0.001). The duration of illness was positively correlated with the number of previous admissions (0.55, 95%CI: 0.51–0.60; *t* = 19.79; *df* = 883; *p* < 0.001). Notably, there were no significant differences observed regarding the rate of compulsory admission.

There were no differences regarding the HoNOS score at admission (*F*(2, 882) = 2.62; *p* = 0.08). However, patients without SUD had a lower HoNOS score at discharge (*F*(2, 882) = 4.06; *p* = 0.02); nonetheless, there were no differences regarding the degree of improvement among the three groups (*F*(2, 882) = 1.64; *p* = 0.19). There were no differences at any point when excluding the HoNOS item 3 assessing alcohol and substance use (for further details see Table 2). Regarding the length of stay (LOS), patients with multiple SUD had a shorter LOS compared to those with no or single SUD (*F*(2, 882) = 9.22; *p* < 0.001). This relationship persisted even after controlling for the age, sex, education, civil status, duration of illness, previous hospitalizations, and antipsychotic treatment. However, for age we found a regression coefficient of − 0.13 (95%CI: − 0.27–0.01); this indicates that for each additional year of age, there is an average decrease of 0.13 days in the length of stay. This negative relationship between age and length of stay was found to be statistically significant (*F*(1, 883) = 93.68, *p* < 0.001). For further information, please refer to Table 2.

The majority of patients (*n* = 567; 64.1%) received treatment with a combination of two antipsychotic medications. Nearly half of the patients (*n* = 434; 49.0%) received at least one agent that is potentially available on the market in a long-acting injectable (LAI) formulation. Available agents in Switzerland are risperidone, paliperidone, aripiprazole, and some first-generation antipsychotics. Olanzapine is not approved as LAI in Switzerland. Despite this potential that almost half of patients could have been directly prescribed their agents as LAI formulation without switching the agent, the overall prescription rate of LAIs was relatively low (*n* = 123, 28.3%). In the group of no SUD, the overall prescription rate of LAI was 9.5% (*n* = 53) compared to 19.9% (*n* = 34) in patients with single SUD and 22.9% (*n* = 36) in patients with multiple SUD (*X* ^2^(2, 434) = 23.62; *p* < 0.001).

Specifically, patients with single SUD had an odds ratio (OR) of 2.62 (95%CI: 1.54–4.43), while those with multiple SUD had an OR of 3.06 (95%CI: 1.80–5.20). After correcting for age, sex, length of stay, previous history (duration of illness, number previous hospitalizations), and severity at admission (HoNOS), patients with single SUD (OR: 1.76; 95%CI 1.05–2.92) and multiple SUD (OR: 2.06; 95%CI: 1.18–3.55) had a higher probability to be prescribed a LAI. Consequently, patients with SUD were much more likely to be prescribed a LAI. For further information, please refer to Table 3.

**Table 3:** Antipsychotic treatment of patients according to the presence of a substance use disorder

	**No SUD**	**Single SUD**	**Multiple SUD**		
	***n*** **= 557**	***n*** **= 171**	*n* **= 157**	Statistic	*p*
	***n (%)***	***n (%)***	***n (%)***		
**Antipsychotic medication**					
Monotherapy	62 (11.1)	17 (9.9)	12 (7.6)	*X* ^2^(2, 885) = 1.64	0.44
Combination (of two)	348 (62.5)	112 (65.5)	107 (68.2)	*X* ^2^(2, 885) = 1.90	0.38
Combination (of three)	147 (26.4)	42 (24.6)	38 (24.2)	*X* ^2^(2, 885) = 0.43	0.80
Antipsychotic available as LAI	265 (47.6)	86 (50.3)	83 (52.9)	*X* ^2^(2, 885) = 1.50	0.47
Antipsychotic prescribed as LAI	53 (9.5)^a^	34 (19.9)^a^	36 (22.9)^a^	*X* ^2^(2, 434) = 23.62	< 0.001
Prescribed LAI antipsychotic	*n* = 53	*n* = 34	*n* = 36		
Aripiprazole	6 (11.3)	5 (14.7)	7 (19.4)	*X* ^2^(2, 123) = 1.13	0.56
Olanzapine	1 (1.9)	0 (0.0)	0 (0.0)	*X* ^2^(2, 123) = 1.33	0.51
Risperidone	4 (7.6)	2 (5.9)	0 (0.0)	*X* ^2^(2, 123) = 2.73	0.25
Paliperidone	37 (69.8)	26 (76.5)	23 (63.9)	*X* ^2^(2, 123) = 1.31	0.51
First-generation antipsychotic	5 (9.4)	1 (2.9)	6 (16.7)	*X* ^2^(2, 123) = 3.75	0.15

^a^No substance use < single and multiple substance use

## Discussion

Our study unveils a significant association between schizophrenia spectrum disorder (SSD) and concurrent substance use disorder (SUD). Specifically, we observed that patients with multiple SUDs typically had shorter hospital stays compared to patients with SSD without SUD or with a single SUD. This might seem paradoxical as firstly patients with one and even multiple comorbid substance disorders are associated with a poorer outcome as evidenced by a higher HONOS score at discharge; however, there are no differences regarding the outcome during the hospitalization, with all groups showing a robust and significant improvement [16]. Furthermore, any differences disappeared after excluding the item measuring alcohol and substance use (HoNOS item 3), although those with substance use tended to have higher scores.

Previous research from the USA reported a relatively short average hospital stay of 9.08 days for patients with schizophrenia [17], which can potentially be further reduced through digitally enhanced relapse prevention [18]. Studies from Switzerland show a median hospital length of stay (HLOS) of 23 days for individuals with SSD [19], aligning with our study’s average stay of 25 days. The general relationship that patients with SSD and comorbid SUD typically have a shorter LOS has already been described in the literature [3–7]. In our study, however, the presence of a single SUD did not significantly alter LOS, while patients with multiple SUDs had noticeably shorter stays. This finding suggests the cumulative effect of multiple SUDs might affect duration of hospitalization.

The lower LOS for patients with multiple SUDs may be due to various factors. One consideration is that about 75% of patients with a single SUD in our study consumed either alcohol or cannabis, while those with multiple SUDs often reported simultaneous use of cocaine or amphetamines. This finding aligns with prior studies demonstrating that comorbid SUD is associated with shorter LOS [3, 4, 6, 7], as well as increased readmission rates and a higher number of hospitalizations [20], leading to a comparable cumulative length of stay [9]. Specifically, the use of cannabis has been generally linked with a reduced HLOS in patients with SSD [6, 7, 21], though this trend may not apply to the initial hospitalization [8]. Likewise, alcohol use has also been associated with shorter stays [4]. Our data corroborate previous findings that stimulants, like cocaine and amphetamines, exert the most significant impact on LOS [3]. It is also worth mentioning that the use of these substances was less likely to occur solely.

There was no significant difference in discharge against medical advice in the subgroups. However, percentage of regular discharge was slightly lower in patients with multiple SUD possibly contributing to the shorter LOS in this subgroup.

In alignment with prior research, our results indicate that cannabis was the most common substance used among patients with SUD (26.2% of cases), followed by alcohol (24.3%), stimulants and cocaine (7.3%), and opioids (5.1%) [22]. Interestingly, our study deviates from previous reports, showing similar rates of cocaine and alcohol use [22]. This finding is noteworthy, given the greater availability and lower cost of alcohol.

Additionally, we found that patients with comorbid SUD were more likely to be prescribed long-acting injectable (LAI) antipsychotics, regardless of the specific medication. This trend may reflect the perception that patients with SUD have lower adherence to oral medications [23] leading to the use of LAIs to ensure continuous psychopharmacological treatment [14].

In our sample, the majority of patients with comorbid SUD were males with limited education beyond regular schooling, and most were single. These demographics suggest lower psychosocial functioning [24]. Moreover, the longer duration of illness by an average younger age observed in patients with SSD and SUD may hint at a shared vulnerability between SSD and SUD, possibly stemming from dysfunction in the meso-cortico-limbic reward circuits [25, 26]. An alternative hypothesis would be that SUD is accelerating the disease onset of SSD without a directly shared vulnerability but as an additional risk factor.

### Limitations

While our study benefits from a naturalistic clinical setting, enhancing the applicability of our findings across a broader psychiatric population, it does present several limitations. First, the details provided differ from those obtained in controlled trials [27]. Thus, we lack of extensive data restricting our ability to calculate important indices as the time of untreated psychosis and the duration of illness. As both could not be extracted from our data, we calculated the time since first admission in general as an approximate measure limiting the reliability of this information on the onset of disease. Since patients have free hospital choice, the cumulative hospital length of stay is restricted, thereby limiting our understanding of the long-term impacts of comorbid SUD on hospital stay durations. Second, the generalizability of our findings to other countries is limited due to potential variations in SUD prevalence and management, influenced by different healthcare systems and contexts. Third, our assessment of SUD primarily relied on patient self-reporting, supplemented occasionally by drug tests. The absence of systematic, standardized drug testing introduces potential inconsistencies and inaccuracies in SUD diagnosis. In addition, although the type of substance use disorder was diagnosed according to the ICD-10 criteria (e.g., dependence, harmful use, intoxication), the pattern and amount of use was not mapped in our clinical data, so that no severity of the disorder could be derived. The present study is therefore limited to assessing the presence of a substance use disorder for each substance class alone. As nicotine use is not routinely diagnosed in our clinic, it is not included in the recording of substance use and it is not possible to draw conclusions on its potential role.

From our data, it is not possible to assign the reason for admission and to generally differentiate whether psychosis or substance disorder was the leading reason for admission.

Concerning HONOS, there is no general reviewer reviewing all HONOS so that there might be a potential inter-rater variability.

Concerning medication, we were not able to reconstruct the prescribed dose from the clinical information system so that this factor cannot be controlled for.

As it is proven that patients with SSD and comorbid SUD have increased risk of psychosocial problems [13], it would be crucial to know how psychosocial factors such as social support or family expressed emotion that might affect the outcome measured by HONOS are not recorded in a standardized way in our clinic, so the sample cannot be characterized concerning these factors and it is not possible to control for them.

The different subgroups cannot be compared regarding antipsychotic treatment efficacy as there is no data of a standardized symptom rating such as PANSS as this is not a routine rating in all patients with SSD in our hospital.

Lastly, our study design inherently precludes us from establishing causality or completely controlling for potential confounding factors. Elements such as the duration of illness and the number of prior hospitalizations might interact with comorbid SUD and affect observed outcomes. Future research using more robust study designs and thorough control for confounders will offer valuable insights into these relationships.

## Conclusions

Our study found that 37.1% of patients with SSD also had a co-occurring SUD, underscoring the high prevalence of this comorbidity. In comparison to those without SUD, patients with this comorbidity exhibited significantly shorter hospital stays and more frequent hospitalizations.

Our analysis is the first to reveal a meaningful relationship between multiple comorbid substance and shorter hospital stay.

We could show that also treatment practices, such as frequent usage of long-acting injectables, are related to the presence of SUD. The increased use of long-acting injectables in patients with SUD requires further study.

In summary, our findings of decreased length of stay, more frequent hospitalizations, and increased use of LAI antipsychotics in patients with comorbid SUDs and SSDs require further investigation. By recognizing and addressing the detrimental consequences of SUD in SSD patients, healthcare providers could improve treatment outcomes and enhance the well-being of this at-risk population.

## Data Availability

The datasets analyzed during the current study are available from the corresponding author on reasonable request due to ethical limitations.

## Abbreviations

HoNOS: Health of the Nation Outcome Scale
ICD-10: International Statistical Classification of Diseases and Related Health Problems, 10th revision
LOS: Length of stay
SD: Standard deviation
SSD: Schizophrenia spectrum disorder
SUD: Substance use disorder

## Declarations

### Declarations

Ethics approval and consent to participate.

The study was conducted in accordance with the Declaration of Helsinki and approved by the Ethics Committee of the Canton of Zurich (BASEC-Nr. Req-2021–00376, approval date: 8 March 2021 and 26 October 2021).

## References

[1] Thornton LK, Baker AL, Lewin TJ, Kay-Lambkin FJ, Kavanagh D, Richmond R, Kelly B, Johnson MP (2012). Reasons for substance use among people with mental disorders. Addict Behav.

[2] National Institute on Alcohol Abuse and Alcoholism (NIAAA) (2000). Health risks and benefits of alcohol consumption. Alcohol Res Health.

[3] Wu HE, Mohite S, Ngana I, Burns W, Shah N, Schneider L, Schmitz JM, Lane SD, Okusaga OO (2015). Hospital length of stay in individuals with schizophrenia with and without cocaine-positive urine drug screens at hospital admission. J Nerv Ment Dis.

[4] Jacobs R, Gutacker N, Mason A, Goddard M, Gravelle H, Kendrick T, Gilbody S (2015). Determinants of hospital length of stay for people with serious mental illness in England and implications for payment systems: a regression analysis. BMC Health Serv Res.

[5] Schmidt LM, Hesse M, Lykke J (2011). The impact of substance use disorders on the course of schizophrenia—a 15-year follow-up study: dual diagnosis over 15 years. Schizophr Res.

[6] Deng H, Desai PV, Mohite S, Okusaga OO, Zhang XY, Nielsen DA, Kosten TR (2019). Hospital stay in synthetic cannabinoid users with bipolar disorder, schizophrenia, or other psychotic disorders compared with cannabis users. J Stud Alcohol Drugs.

[7] Johnson JM, Wu CY, Winder GS, Casher MI, Marshall VD, Bostwick JR (2016). The effects of cannabis on inpatient agitation, aggression, and length of stay. J Dual Diagn.

[8] Manrique-Garcia E, Zammit S, Dalman C, Hemmingsson T, Andreasson S, Allebeck P (2014). Prognosis of schizophrenia in persons with and without a history of cannabis use. Psychol Med.

[9] Florentin S, Rosca P, Raskin S, Bdolah-Abram T, Neumark Y (2019). Psychiatric hospitalizations of chronic psychotic disorder patients with and without dual diagnosis, Israel, 1963–2016. J Dual Diagn.

[10] Barnes TR, Mutsatsa SH, Hutton SB, Watt HC, Joyce EM (2006). Comorbid substance use and age at onset of schizophrenia. Br J Psychiatry.

[11] Loyal JP, Lavergne MR, Shirmaleki M, Fischer B, Kaoser R, Makolewksi J, Small W (2023). Trends in involuntary psychiatric hospitalization in British Columbia: descriptive analysis of population-based linked administrative data from 2008 to 2018. Can J Psychiatry.

[12] Fazel S, Yu R (2011). Psychotic disorders and repeat offending: systematic review and meta-analysis. Schizophr Bull.

[13] Compton MT, Weiss PS, West JC, Kaslow NJ (2005). The associations between substance use disorders, schizophrenia-spectrum disorders, and axis IV psychosocial problems. Soc Psychiatry Psychiatr Epidemiol.

[14] Reymann S, Schoretsanitis G, Egger ST, Mohonko A, Kirschner M, Vetter S, Homan P, Seifritz E, Burrer A (2022). Use of long-acting injectable antipsychotics in inpatients with schizophrenia spectrum disorder in an academic psychiatric hospital in Switzerland. J Pers Med.

[15] Wing J, Curtis R, Beevor A (1994). ‘Health of the Nation’: measuring mental health outcomes. Psychiatr Bull.

[16] Egger ST, Bobes J, Theodoridou A, Seifritz E, Vetter S (2020). Assessing the severity of psychiatric disorders using the Health of the Nation Outcome Scales: an equipercentile linking analysis. Aust N Z J Psychiatry.

[17] Chen E, Bazargan-Hejazi S, Ani C, Hindman D, Pan D, Ebrahim G, Shirazi A, Banta JE (2021). Schizophrenia hospitalization in the US 2005–2014: examination of trends in demographics, length of stay, and cost. Medicine (Baltimore).

[18] Homan P, Schooler NR, Brunette MF, Rotondi A, Ben-Zeev D, Gottlieb JD (2022). Relapse prevention through health technology program reduces hospitalization in schizophrenia. Psychol Med.

[19] Lay B, Nordt C, Rössler W (2007). Trends in psychiatric hospitalisation of people with schizophrenia: a register-based investigation over the last three decades. Schizophr Res.

[20] Busch AB, Epstein AM, McGuire TG, Normand SL, Frank RG (2015). Thirty-day hospital readmission for Medicaid enrollees with schizophrenia: the role of local health care systems. J Ment Health Policy Econ.

[21] Williams SR, Agapoff JRT, Jalan D, Hishinuma ES, Kida LE (2021). Psychiatric hospitalization and length of stay differences in cannabis users and non-users with a primary discharge diagnosis of schizophrenia or schizoaffective disorder. Subst Use Misuse.

[22] Hunt GE, Large MM, Cleary M, Lai HMX, Saunders JB (2018). Prevalence of comorbid substance use in schizophrenia spectrum disorders in community and clinical settings, 1990–2017: systematic review and meta-analysis. Drug Alcohol Depend.

[23] Jónsdóttir H, Opjordsmoen S, Birkenaes AB, Simonsen C, Engh JA, Ringen PA, Vaskinn A, Friis S, Sundet K, Andreassen OA (2013). Predictors of medication adherence in patients with schizophrenia and bipolar disorder. Acta Psychiatr Scand.

[24] Lin D, Kim H, Wada K, Aboumrad M, Powell E, Zwain G, Benson C, Near AM (2022). Unemployment, homelessness, and other societal outcomes in patients with schizophrenia: a real-world retrospective cohort study of the United States Veterans Health Administration database: societal burden of schizophrenia among US veterans. BMC Psychiatry.

[25] Khokhar JY, Dwiel LL, Henricks AM, Doucette WT, Green AI (2018). The link between schizophrenia and substance use disorder: a unifying hypothesis. Schizophr Res.

[26] Kirschner M, Rabinowitz A, Singer N, Dagher A (2020). From apathy to addiction: insights from neurology and psychiatry. Prog Neuropsychopharmacol Biol Psychiatry.

[27] Smeets HM, de Wit NJ, Hoes AW (2011). Routine health insurance data for scientific research: potential and limitations of the Agis Health Database. J Clin Epidemiol.

